# C2c: Predicting Micro-C from Hi-C

**DOI:** 10.3390/genes15060673

**Published:** 2024-05-23

**Authors:** Hao Zhu, Tong Liu, Zheng Wang

**Affiliations:** Department of Computer Science, University of Miami, 330M Ungar Building, 1365 Memorial Drive, Coral Gables, FL 33124-4245, USA; hxz527@miami.edu (H.Z.); tong.liu@miami.edu (T.L.)

**Keywords:** Micro-C, predicting Micro-C from Hi-C, deep learning, residue network

## Abstract

Motivation: High-resolution Hi-C data, capable of detecting chromatin features below the level of Topologically Associating Domains (TADs), significantly enhance our understanding of gene regulation. Micro-C, a variant of Hi-C incorporating a micrococcal nuclease (MNase) digestion step to examine interactions between nucleosome pairs, has been developed to overcome the resolution limitations of Hi-C. However, Micro-C experiments pose greater technical challenges compared to Hi-C, owing to the need for precise MNase digestion control and higher-resolution sequencing. Therefore, developing computational methods to derive Micro-C data from existing Hi-C datasets could lead to better usage of a large amount of existing Hi-C data in the scientific community and cost savings. Results: We developed C2c (“high” or upper case C to “micro” or lower case c), a computational tool based on a residual neural network to learn the mapping between Hi-C and Micro-C contact matrices and then predict Micro-C contact matrices based on Hi-C contact matrices. Our evaluation results show that the predicted Micro-C contact matrices reveal more chromatin loops than the input Hi-C contact matrices, and more of the loops detected from predicted Micro-C match the promoter–enhancer interactions. Furthermore, we found that the mutual loops from real and predicted Micro-C better match the ChIA-PET data compared to Hi-C and real Micro-C loops, and the predicted Micro-C leads to more TAD-boundaries detected compared to the Hi-C data. The website URL of C2c can be found in the Data Availability Statement.

## 1. Introduction

Understanding how the chromatin folds from individual nucleosomes to entire chromosome territories is very important in exploring the genome process [1]. Chromosome conformation capture technology and its genome-wide derivatives have revolutionized the spatial organization of chromatin folding [2]. These methods use proximity ligation of cross-linked genomic loci to estimate the number of interactions between genomic regions across the chromosome. The main difference between the 3C-based technologies is their scope. For example, 3C experiments [2] mainly detect the interactions between two specific fragments. However, Hi-C experiments [3] quantify the interactions between all possible pairs of fragments simultaneously. Based on a genome-wide scope technique like Hi-C, we can characterize some special chromosome structures across the whole genome, such as topologically associating domains [4] and chromatin loops [5]. 

TADs are defined as local genomic domains within which genomic loci have more frequent interactions than with loci outside the domains. These structures appear as square boxes along the diagonal of 3D contact maps [4]. Chromatin loops are defined as pairs of genomic loci that exist far at a genomic distance but are close at spatial proximity by the loop extrusion [5]. More and more evidence verifies that CTCF and cohesion regulate TAD formation via the loop extrusion mechanism [6]. Chromatin loops appear as sharp corner peaks or dots in contact maps [7].

A lot of research has reported that transcriptional regulation is influenced by these TADs and loops [7]. The disruption of these structures will cause certain diseases [8]. Although the TADs and loops can be detected from the Hi-C contact maps, conventional Hi-C can only identify a limited number and size of these structures due to the limitation of the kilobase to ten kilobase length of digesting restriction enzymes. In mammals, how chromatin is folded at finer scales remains largely unexplored.

The high-resolution Hi-C data that can detect the chromatin features below the level of TADs will significantly increase our understanding of gene regulation. Micro-C, a version of Hi-C that includes a micrococcal nuclease digestion step to look at the interactions between pairs of nucleosomes, was invented to overcome the resolution limitations of Hi-C [9,10,11]. Micro-C measures the contacts between pairs of crosslinked nucleosomes, which can investigate the chromatin organization at a resolution of 100 bp. In a previous study [12], the researchers discovered the principles of chromatin folding below the scale of TADs by employing the Micro-C for mouse embryonic stem (mES) cells at high resolution (up to 100 bp). From the side-by-side comparison of their published Micro-C and current highest-depth Hi-C data, Micro-C data recapitulated all the reported chromatin structures, such as TADs and loops, with high reproducibility scores and comparable data quality [12].

Although Micro-C allows us to investigate more detailed chromatin structures that may be functionally relevant to gene regulation, Micro-C experiments are more technically challenging than Hi-C due to the need for precise MNase digestion control and higher-resolution sequencing [9,10]. Therefore, developing computational methods to derive Micro-C data from existing Hi-C datasets could lead to greater efficiency and cost savings.

Previous studies [13,14] have released computational methods like HiCNN and HiCPlus to enhance the resolutions of Hi-C data, which use convolutional neural networks (ConvNet). They used the learned models to study the repeatable patterns between the low-resolution and high-resolution Hi-C data and finally obtained a robust model for predicting the high-resolution Hi-C data from the low-resolution data. However, it is worth noting that these tools were all trained with the high-resolution Hi-C contact matrices as the target values instead of the Micro-C contact matrices. Although some Hi-C experiments were claimed to achieve Hi-C at 1 kb resolution [5], it is generally not comparable to the resolution that Micro-C can achieve. Furthermore, because Hi-C and Micro-C use different ways to cut the genome, some of the loops are lost even in high-resolution Hi-C. Therefore, our tool is different from Hi-C resolution enhancement.

In our study, we developed computational methods to accurately predict Micro-C data based on Hi-C data. We employed a residual neural network [15], customizing the residual block. Our evaluation results show that the predicted Micro-C contact matrices can better reveal chromatin loops and TAD boundaries than the input Hi-C contact matrices. Our tool also showed better performance compared to HiCNN, a state-of-the-art tool for Hi-C resolution enhancement.

## 2. Materials and Methods

### 2.1. Micro-C Data and Hi-C Data

We downloaded the Micro-C data of mouse embryonic stem cells from the GEO (Gene Expression Omnibus) database with accession number GSE130275. We selected the “.mcool” file and used the COOLER package 0.9.2 [16] in Python to extract the contact matrix for different chromosomes at 1 kb and 5 kb resolutions. The Hi-C data is from the GEO database with accession number GSE96107. This data is the same cell type as Micro-C data, and both used the mm10 reference genome for mapping. We downloaded “fastq” files of all the mouse embryonic stem cells and converted them into 1 kb and 5 kb COOLER files.

For training 5 kb resolution data, we used all the chromosomes for training, validation, and blind testing. We trained a total of six different models. For each model, we left one chromosome for validation, one chromosome for blind testing, and the rest for training. We randomly selected and used chromosomes 1, 4, 7, 10, 14, and 17 for the blind testing of these six models. Except for the model for blind testing on chromosome 14, we set chromosome 14 for validation and the rest of the chromosomes for training. For the model that was blind-tested on chromosome 14, we used chromosome 17 for validation and all the other chromosomes for training.

For training using 1 kb resolution data, we used mouse embryonic stem cells from chromosome 1 to chromosome 13 for training, chromosome 17 for validation, and the rest chromosomes as blind test datasets: chromosomes 14, 15, 16, 18, and 19.

We also downloaded the Micro-C and Hi-C data from human embryonic stem cells (hES) [17] to test our deep learning model that was trained on mES cells. We used chromosomes 10 and 17 for blind testing at a 5 kb resolution.

### 2.2. Contact Matrix Generation

We generated the training data at 1 kb and 5 kb resolutions, which means we trained two separate sets of models for the data at two different resolutions. The reason why we selected these two resolutions is that these are the two highest resolutions that can detect clear, distinct numbers of loops and TADs (only a few structures can be detected at 200 bp or higher resolutions) [12].

For training and validation data, we set a 200 × 200 matrix as the unit matrix and pushed it into the network. For 1 kb resolution, we extracted the unit matrices within the range of 2 Mb regions (2000 beads) before and after the diagonal in the horizon direction in the contact map. A sliding pattern was applied, with 100 overlapping, which resulted in different 200 × 200 matrices having overlapping regions. In the vertical direction, we excluded the first and last 10 Mb sections of the chromosomes and applied the same processing method as in the horizontal direction. For the 5 kb resolution, we extended the range from 2 Mb to 10 Mb (2000 beads). The rest of the approaches were the same with a 1 kb resolution.

Since the original values of Hi-C and Micro-C contacts are close to zero, to balance the distribution of the values, we normalized the Hi-C and Micro-C values. Specifically, we first set a cutoff of 0.05 to set the values larger than 0.05 equal to 0.05. Then, we employed the following expression for normalizing the values: ${log}_{10}(1+9\times c/0.05)$, where c is the original contact value, and ‘0.05’ here is the max value in the contact map after cutoff.

### 2.3. Customizing Residual Neural Network Architecture

We developed a Residual Neural Network (ResNet) comprising three main components. The network inputs are Hi-C contact submatrices shaped as n × 1 × 200 × 200, where n represents the batch size, “1” indicates the input channel size of the initial block, and 200 × 200 defines the matrix size. The first component consists of an initial block utilizing a 2D convolutional layer to increase the hidden dimension from one to 64, accompanied by a batch normalization layer and a ReLU layer.

The second component encompasses 24 residual blocks, each starting with a 2D convolution layer, followed sequentially by a 2D batch normalization layer, a ReLU layer, another 2D convolution layer, and an additional 2D batch normalization layer. The output of this sequence is then added back to the input of the residual block, ending in a final ReLU layer. As data traverses through all the residual blocks, the original input is merged with the output transformed by each residual block, maintaining the data dimensions at a constant 64 in every block.

The third part employs a single 2D convolutional layer to reduce the dimensionality from 64 to one. The outputs from the network are the predicted Micro-C submatrices with the same shape as the inputs.

### 2.4. Training, Validation, and Blind Testing

We implemented our network using PyTorch. For optimization, we chose the Adam optimizer. The learning rate scheduler employed was ‘ReduceLROnPlateau’, configured with the parameters ‘min’, ‘factor = 0.5’, and ‘patience = 5’. We tried four different learning rates (0.1, 0.01, 0.001, and 0.0001), five different batch sizes (1, 4, 8, 16, and 32), and five different numbers of residual blocks (4, 8, 12, 16, 20, 24). The combination of a 0.001 learning rate, a batch size of 32, and 24 residual blocks yielded the best results on our validation dataset. This configuration was then used in our final model for the blind test. The effectiveness of our models was primarily judged by the lowest validation loss they achieved. We used the mean squared error as the loss function for all models. Our training was conducted on an NVIDIA A100 GPU with 40 GB of memory. To train the best-performing model, it took approximately 24 h and consumed around 32 GB of GPU memory.

### 2.5. Evaluation Methods

To analyze the discrepancy between the predicted and real Micro-C contact maps, we employed the methods below to identify chromatin loops and boundaries from the maps.

We used Mustache [18] for multi-scale detection of chromatin loops based on Hi-C and Micro-C contact maps at high resolutions (1 kb and 5 kb). We ran Mustache with a normalized contact map, a bias vector (set to 1000), and a *p*-value cutoff of 0.05 to identify the loops in the Hi-C, Micro-C, and predicted Micro-C maps.

To test the reliability of these loops, we employed the aggregate peak analysis (APA) method [5]. This method assesses the overall enrichment of a set of putative two-dimensional loops. By averaging Hi-C, real Micro-C, or predicted Micro-C maps, we created pileup contact maps with each loop located in the center. Specifically, we first retrieved local matrices centered on loop positions calculated by Mustache. The windows used were +/−25 kb for both 5 kb and 1 kb resolutions before and after each anchor of a loop. To neutralize the effects of various genomic distances, we used the observed over the expected numbers of interactions when conducting the APA analysis. These pileup procedures were conducted using the cooltools 0.5.4 [19] package in Python.

For APA plots, we determined focal enrichment at the center pixel by calculating the APA score—the ratio of values in the center bin to the average number of reads in a 15 kb × 15 kb window in the lower-left corner.

To try to find whether the loops detected from Hi-C, predicted Micro-C, and real Micro-C correlate with ChIA-PET, we also conducted an APA measurement based on pileups on the mES CTCF ChIA-PET data from the ENCODE database (experiment ENCSR214BLK).

Given that loops are often enriched for promoters and enhancers, we identified promoter–enhancer loops among those detected in our contact maps. We utilized ENCODE-combined HMM segmentation [20] to identify enhancer–promoter loops that have a transcription start state on one loop anchor and either an enhancer or active promoter state on the other.

We used cooltools [19] to identify TAD boundaries in Hi-C, predicted Micro-C, and real Micro-C contact maps, setting the boundary window size to 0.6 Mb for 500 kb resolution.

### 2.6. HiCNN Comparison

We used the pre-trained HiCNN model to enhance the same 5 kb resolution Hi-C data as C2c used for blind testing. When generating the input Hi-C data, we also set the same cutoff of 0.05 for Hi-C contacts. We did not use HiCNN to generate 1 kb data because it was designed to enhance data at 10 kb resolution; 1 kb is too large for HiCNN to process effectively.

When evaluating the output of HiCNN, we employed the same parameters and methods as those used for 5k C2c-predicted Micro-C to ensure a fair comparison. The results of this comparison are detailed in the subsequent sections of the results.

## 3. Results

### 3.1. C2c-Predicted Micro-C Contact Maps

**Figure 1:**
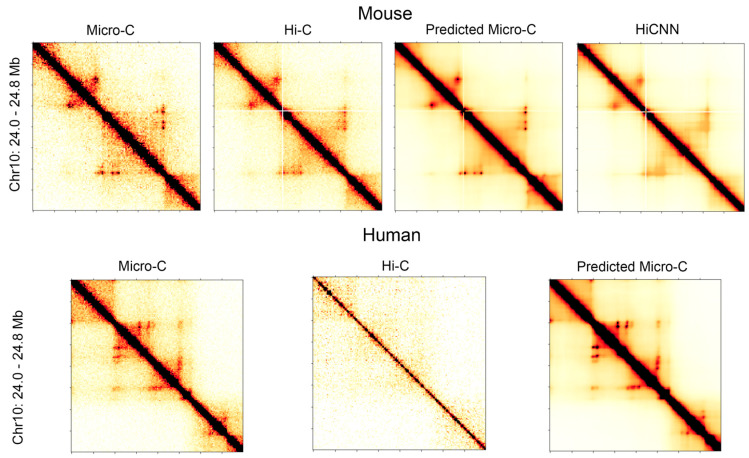
Heatmaps of the contact maps for chromosome 10 from 24.0 to 24.8 Mb at 5 kb resolution. The heatmaps in the first row are for the real Micro-C, Hi-C, C2c-predicted Micro-C, and HiCNN enhanced Hi-C for mES cells. The heatmaps in the second row are for hES cells.

Figure 1 shows a side-by-side comparison of the heatmaps for the genomic region of 24.0 Mb to 24.8 Mb in chromosome 10 at 5 kb resolution. The heatmaps in the first row are for the real Micro-C, Hi-C, C2c-predicted, and HiCNN enhanced Hi-C for mouse embryonic stem cells, and the heatmaps in the second row are for the real Micro-C, Hi-C, and C2c-predicted Micro-C for human embryonic stem cells. From the contact maps of the mouse cell, it can be found that the loops in the predicted Micro-C contact map are more distinct and obvious compared to both the Hi-C contact maps and the HiCNN-enhanced Hi-C contact maps, demonstrating the enhancement of the loop regions of C2c. For human cells, the Hi-C data have a much lower sequencing depth compared to the Hi-C data of mouse ES cells, but C2c still makes it more closely resemble Micro-C.

### 3.2. APA Scores on Loop Regions

**Figure 2:**
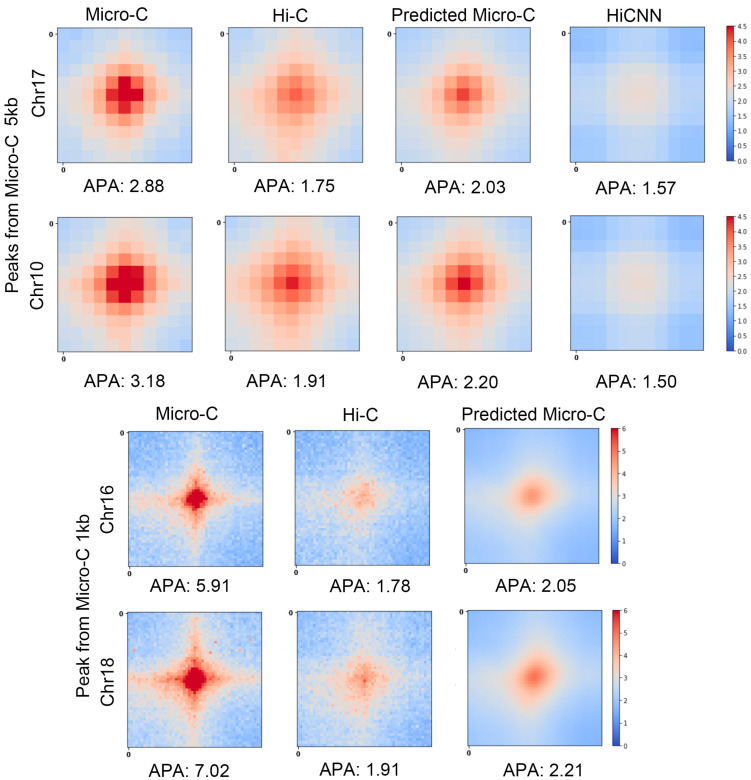
The heatmaps of the averaged contact maps of real Micro-C, Hi-C, C2c-predicted Micro-C, and HiCNN-enhanced HiC that centered at the loops from the real Micro-C data. The APA score is shown at the bottom of each heatmap. The top two rows have a 5 kb resolution, and the bottom two have a 1 kb resolution, all based on mES cells.

To validate the accuracy of our predicted Micro-C interactions, we used APA measurement on the real Micro-C contact maps, Hi-C contact maps, C2c-predicted Micro-C maps, and the HiCNNN-enhanced HiC maps. Figure 2 shows the APA scores along with the heatmaps of the averaged contact matrices centered at the loops at 5 kb and 1 kb resolutions for mES cells. We can find that the APA scores for predicted Micro-C are higher than the ones for Hi-C and HiCNN-enhanced Hi-C. For chromosome 17, the APA score of the predicted Micro-C, which is 2.03, is the closest to the APA score of the real Micro-C, which is 2.88.

At a 1 kb resolution, the Hi-C heatmap exhibits a very pale central region with dispersed red points, indicating weaker interaction signals. As for the predicted Micro-C, this central area becomes significantly more pronounced and now closely resembles the corresponding part of the Micro-C heatmaps. This proves that our model can effectively enrich Hi-C contact maps to more closely approach the quality observed in real Micro-C data.

We performed APA measurements on additional chromosomes, including four extra chromosomes at 5 kb resolution and three extra chromosomes at 1 kb resolution. These results are presented in Supplementary Figure S1. The findings across these analyses further substantiate our conclusion.

**Figure 3:**
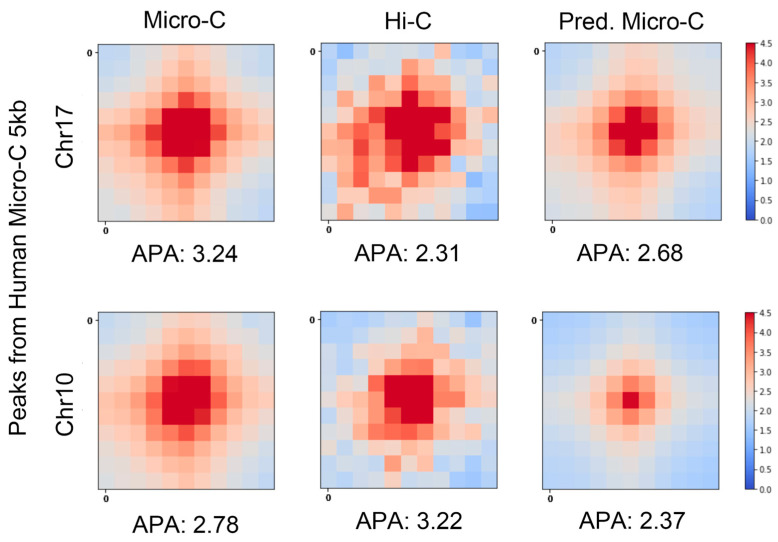
APA scores based on hES cells at 5 kb resolution. The first row shows the heatmaps of chr17 of the averaged contact maps of real Micro-C, Hi-C, and C2c-predicted Micro-C that are centered at the loops from the real Micro-C data. The APA score is shown at the bottom of each heatmap. The second row is similar to the first row, but for chr10.

We also performed the APA measurements on hES cells at 5 kb resolution, as shown in Figure 3. The real Micro-C and C2c-predicted Micro-C heatmaps show strong central interactions and have more similar APA scores on chromosome 7. We found that the APA score of Hi-C in chromosome 10 is higher than C2c-predicted Micro-C. In the next section, we will show that the number of loops detected on Hi-C data was much smaller than the number of loops detected on C2c-predicted Micro-C data. This may indicate that only a few highly intensive loops were detected from Hi-C data.

### 3.3. Overall Statistics of Loops

**Figure 4:**
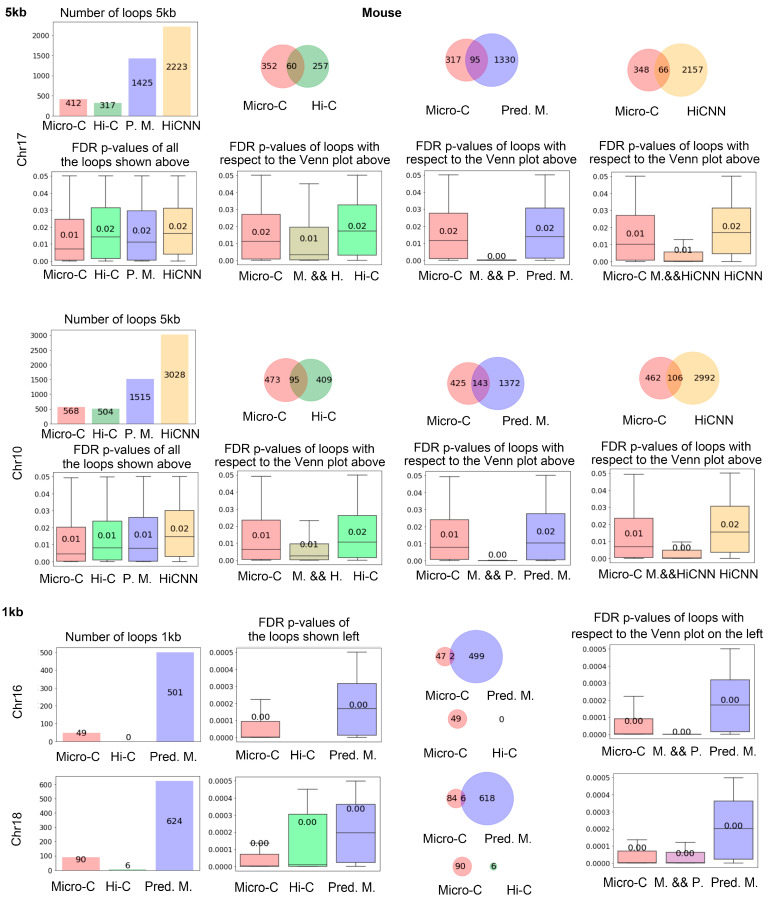
The loop analysis of mES cells at 5 kb and 1 kb resolution. In the first row, the numbers of total loops detected for chr17 at 5 kb resolution are shown, including the loops from the real Micro-C, Hi-C, C2c-predicted Micro-C, and HiCNN-enhanced Hi-C. The three Venn figures on the right are the Venn plots of the loops. The second row displays the FDR *p*-values calculated by Mustache for the corresponding loops. Each box plot figure matches the above figure, showing the values of the loops from the independent data type or intersections of the data types. The third and fourth rows are the results for chr10 at 5 kb resolution, similar to the top two rows. The bottom two rows show analysis results at 1 kb resolution.

Figure 4 shows the number of loops identified by Mustache based on real Micro-C, Hi-C, predicted Micro-C, and HiCNN-enhanced Hi-C from mES cells. The bar plot on the top left displays the quantity of these loops, illustrating that real Micro-C data result in a higher number of loops compared to Hi-C data, and our predicted Micro-C data lead to even more loops on chr17 at 5 kb resolution. We found that HiCNN resulted in even more loops. The three diagrams on the right of the first row show the Venn plots for the loop detected from different types of data. From the plots, we can find that the predicted Micro-C leads to more loops that are consistent with the real Micro-C than Hi-C and HiCNN-enhanced Hi-C. It also means that even though HiCNN-enhanced Hi-C can detect more loops, fewer of these loops match the Micro-C loops.

The four box plots under the first row show the false discovery rate (FDR) *p*-values output from the loop-calling tool Mustache, which provides a measure of statistical significance in loop identifications. The left figure displays the FDR *p*-values of all of the loops detected in each type of data. We can find that real Micro-C loops have the lowest FDR *p*-values, followed by the C2c-predicted Micro-C, Hi-C, and HiCNN-enhanced Hi-C, which indicate that C2c can detect the loops with slightly higher significance than Hi-C and HiCNN-enhanced Hi-C.

The three figures on the right show the FDR *p*-values for the Venn diagrams on top of them, respectively. It can be found that the mutual loops detected by both real Micro-C and C2c-predicted Micro-C have a lower *p*-value compared to the mutual loops between HiCNN and real Micro-C, and the additional 1330 loops detected by C2c but not found in real Micro-C data were also statistically significant. In the next section, we will demonstrate that C2c-predicted Micro-C data lead to more loops that match the known promoter–enhancer interactions compared to real Micro-C. Together, these may indicate that the deep learning algorithm of C2c rebuilt many loops that were not captured by the real Micro-C data. The figures in the third and fourth rows were from chr10, which indicated similar conclusions.

At the 1 kb resolution (rows 5 and 6 in Figure 4), the input Hi-C data result in a very small number of loops, and the number of loops detected from real Micro-C data is also much smaller than the one at the 5 kb resolution. Compared to using a *p*-value cutoff of 0.05 for running Mustache at 5 kb resolution, we set the *p*-value cutoff as 0.0005 for 1 kb resolution to include only the most significant loops. Furthermore, due to the small number of loops identified by the 1 kb contact map, we allowed a bin error of +/−1000 bp at each end of the pair-end when determining whether two loops are identical. For the following finding of the consistent enhancer–promoter loops at 1 kb resolution, we used the same criteria. It can be found that C2c-predicted Micro-C leads to many more loops compared to real Micro-C and Hi-C, and the loops detected from C2c-predicted Micro-C are statistically significant.

**Figure 5:**
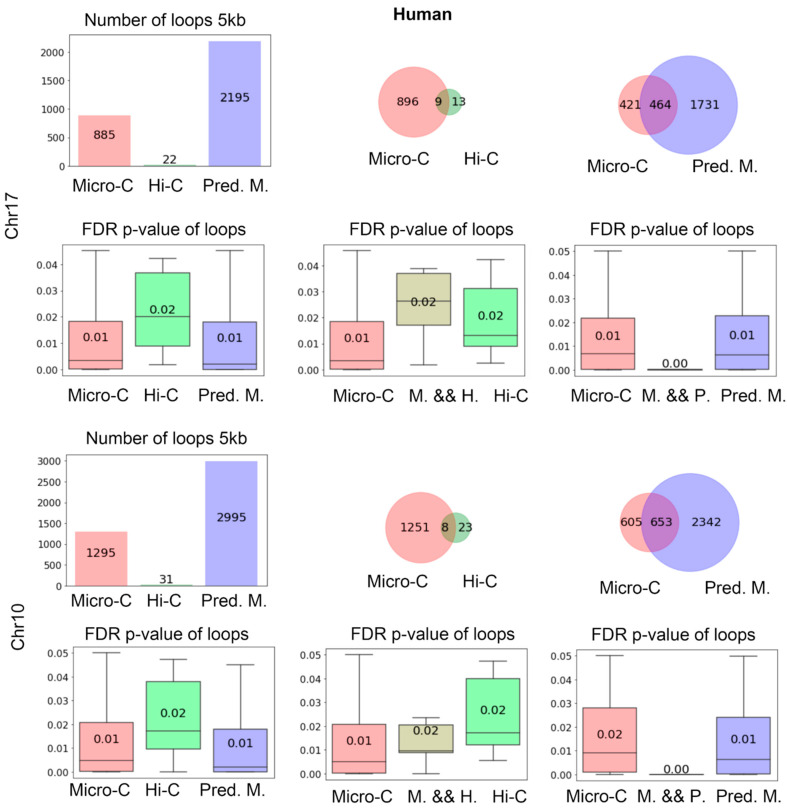
The loop analysis of hES cells at 5 kb resolution. In the first row, the numbers of total loops detected for chr17 at 5 kb are shown, including the loops from real Micro-C, Hi-C, and C2c-predicted Micro-C. The two Venn diagrams on the right show the intersections of loops. The second row displays the FDR *p*-values calculated by Mustache for the corresponding loops. Each box plot figure matches the above figure. The third and fourth rows are the results for chr10, similar to the top two rows.

We present the evaluation results for hES cells at 5 kb resolution in Figure 5. We can draw similar conclusions from the results in human cells as in mouse cells. This further verified that C2c is robust and can be effectively applied across different species since the deep learning model was trained on mouse data.

**Figure 6:**
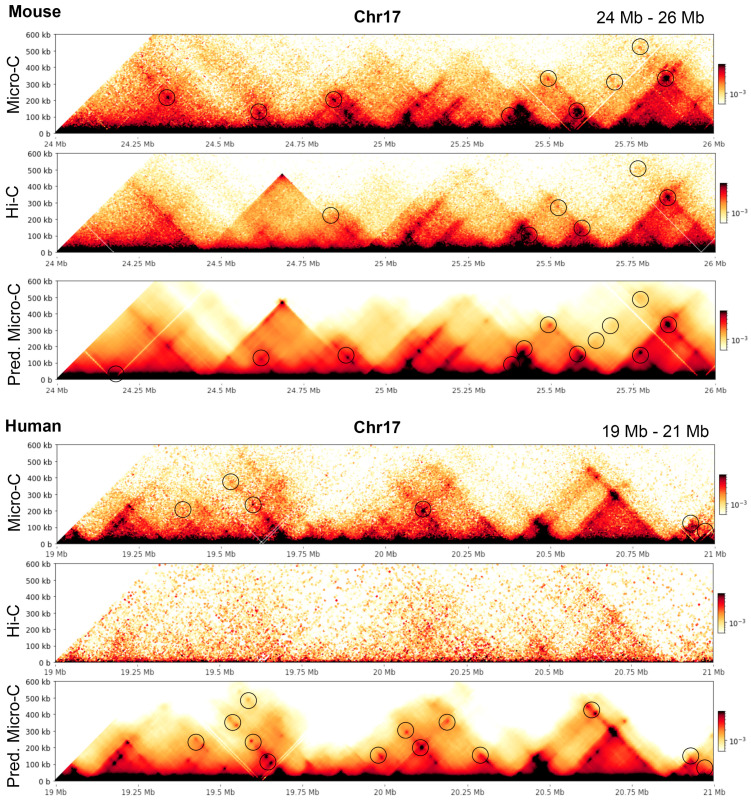
Heatmaps with the loops detected highlighted in 5 kb mES and hES data.

To give examples illustrating the conclusions, we extracted a region in mouse and human chromosomes 17 (24–26 Mb in mouse and 19–21 Mb in human) at 5 kb resolution and highlighted the loops recognized by Mustache with circles; see Figure 6.

### 3.4. Matching the Detected Loops with Enhancer–Promoter Interactions

**Figure 7:**
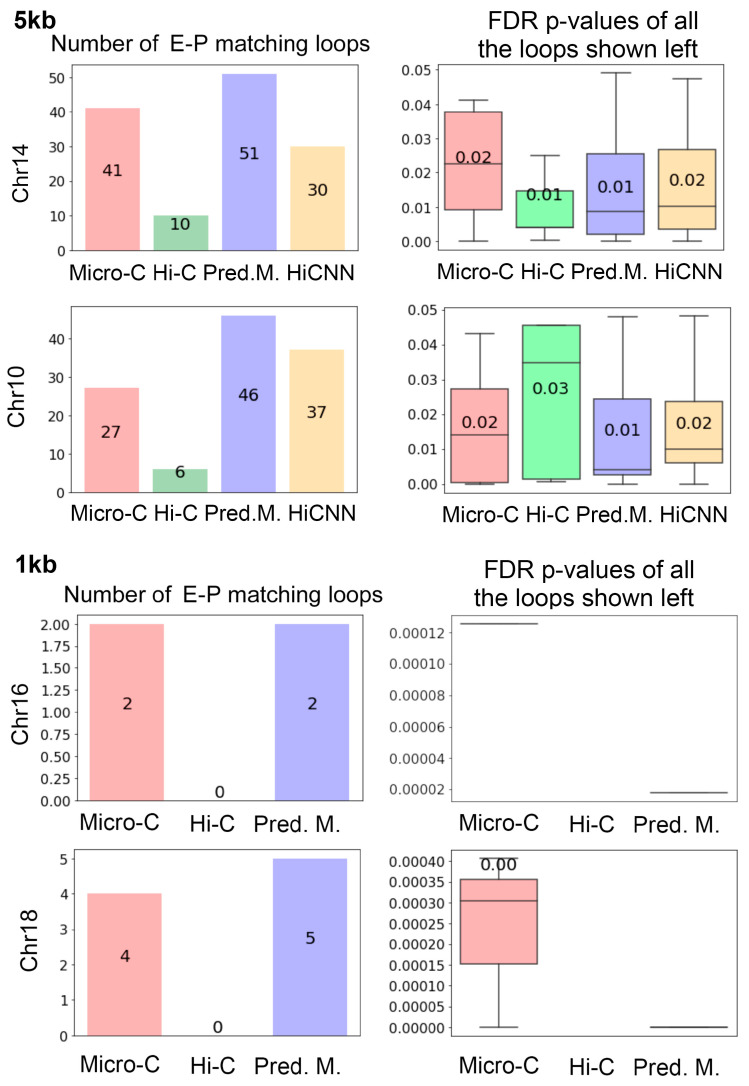
The number of total enhancer–promoter matching loops detected for Micro-C, Hi-C, C2c-predicted Micro-C, and HiCNN-enhanced Hi-C, together with the FDR *p*-values generated from the loop calling tool Mustache.

To verify the validity of the greater number of loops resulting from predicted Micro-C, we employed the property that loops are usually enriched for promoters and enhancers. The bar plots in Figure 7 represent the quantities of loops that match enhancer–promoter interactions on mES cells at 5 kb and 1 kb resolutions. The real Micro-C contains more enhancer–promoter matching loops than those from Hi-C, and the C2c-predicted Micro-C is associated with even more enhancer–promoter matching loops than the real Micro-C. Meanwhile, the HiCNN-enhanced Hi-C captured fewer enhancer–promoter matching loops than the C2c-predicted Micro-C. Supplementary Figure S2 includes additional results on more chromosomes at two different resolutions, which also demonstrate similar patterns.

### 3.5. Cross-Validation with ChIA-PET

**Figure 8:**
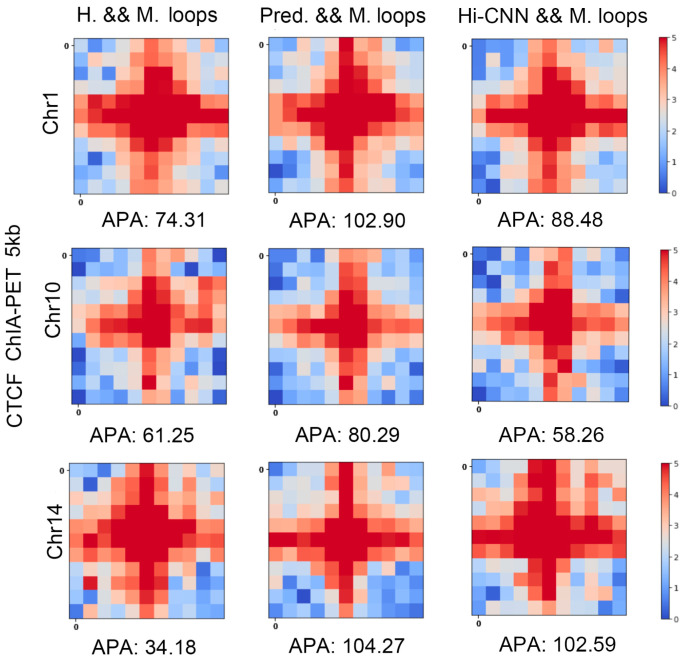
The heatmaps of the averaged ChIA-PET contact maps at 5 kb resolution centered at the loops from Hi-C and Micro-C mutual loops, C2c-predicted Micro-C and real Micro-C mutual loops, and HiCNN-enhanced Hi-C and Micro-C mutual loops. The APA score is shown at the bottom of each heatmap.

We also cross-validated the predicted loops with another experiment, that is, the CTCF ChIA-PET in mES cells. In Figure 8, “H. & M. loops” indicates the heatmap of averaged ChIA-PET contact matrices centered at the mutual loops of Hi-C and real Micro-C. “Pred. & M. loops” is the same type of plot but for the mutual loops of predicted Micro-C and real Micro-C. The APA scores from the mutual loops of Hi-C and Micro-C are much smaller than the ones of predicted Micro-C and real Micro-C, which indicates the mutual loops between predicted loops and real Micro-C loops better fit CTCF ChIA-PET. We can also find that the APA scores from the mutual loops of HiCNN-enhanced Hi-C and Micro-C are smaller than the ones of C2c-predicted Micro-C and Micro-C, which means that the loops detected from C2c-predicted Micro-C better fit CTCF ChIA-PET.

### 3.6. TAD Boundaries

**Figure 9:**
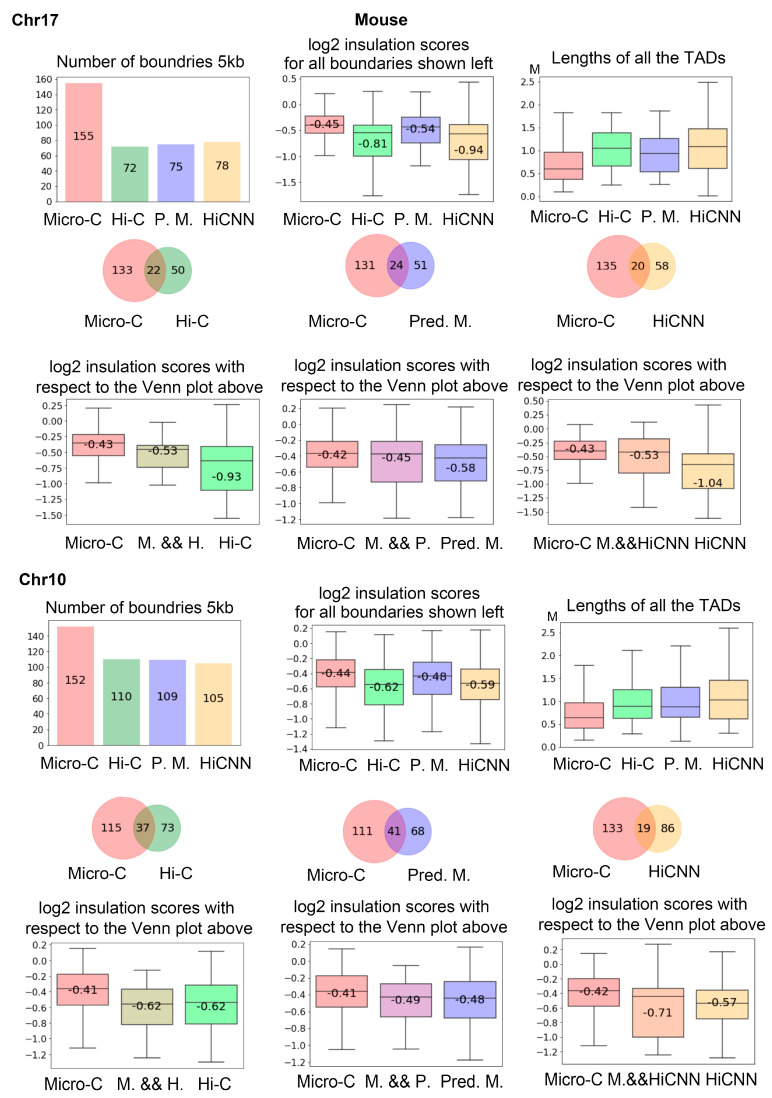
The measurements of the TAD boundaries that were identified from the mES cells at 5 kb resolution. The first row shows the number of total TAD boundaries detected for chr17, including the boundaries from the real Micro-C, Hi-C, C2c-predicted Micro-C, and HiCNN-enhanced Hi-C. On the right, it shows the distributions of the log2 insulation scores of the corresponding boundaries. In the far right, it shows the length of TADs from the different data types. The three Venn figures in the second row are the intersections of boundaries from Micro-C and Hi-C, from Micro-C and C2c-predicted Micro-C, and from Micro-C and HiCNN-enhanced Hi-C. The box plots in the third row match the above figures, showing the insulation scores of the boundaries from the independent data type or intersections of the data types. The bottom three rows are similar to the top three rows, but for chr10.

Figure 9 shows the analysis of the TAD boundaries identified in different types of data on mES cells at 5 kb resolution. From the first row, we can see that real Micro-C data leads to more TAD boundaries that have higher insulation, scores indicating weaker boundaries, and the TADs detected from real Micro-C data are generally smaller.

The Venn plots and box plots in the second and third rows display intersection status and the log2 insulation scores for the boundaries. The predicted Micro-C leads to more mutual TAD boundaries with real Micro-C in comparison to between Hi-C and real Micro-C and between HiCNN-enhanced Hi-C and real Micro-C. The fourth to sixth rows in Figure 9 are similar to the first to third rows, but for chr10.

**Figure 10:**
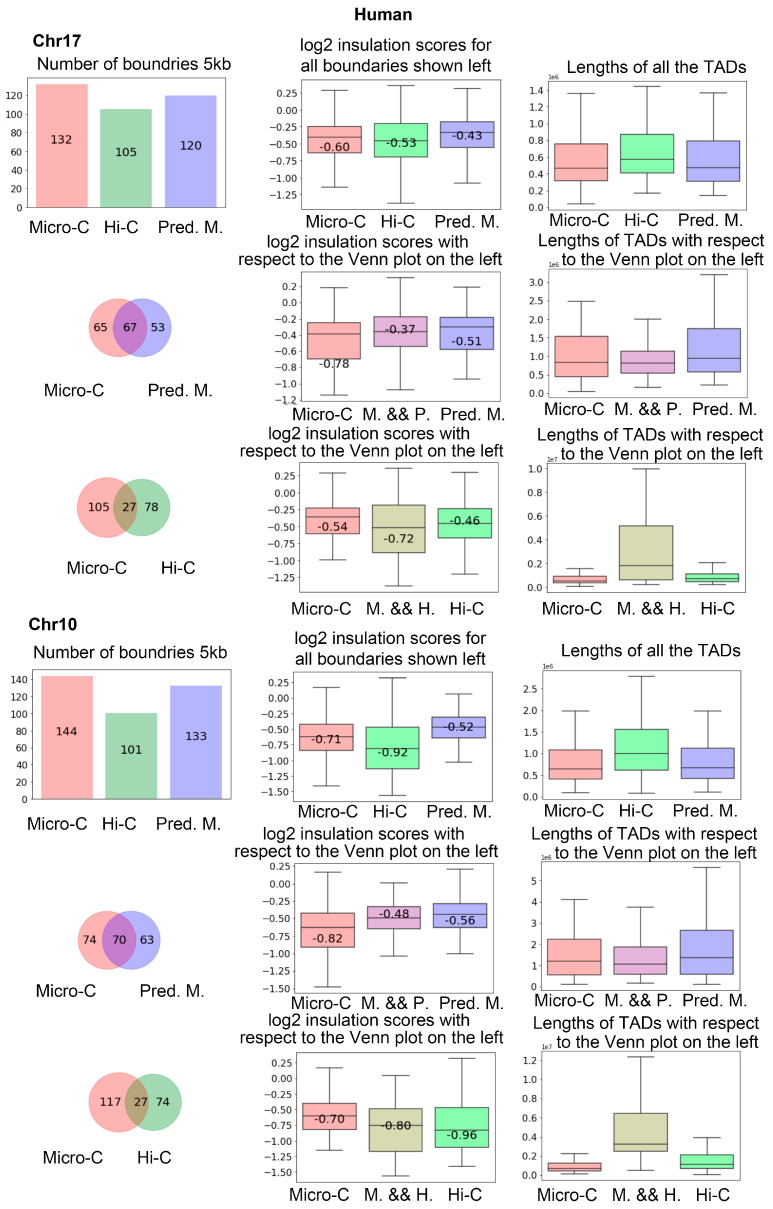
The analysis of the TADs boundaries identified from the hES cells at 5 kb resolution. The first row shows the results for all the boundaries detected on chr17. The second and third rows show the analysis as Venn diagrams, together with the log2 insulation scores. The rest of the figure is for chr10.

We also performed the same analysis on human data. In Figure 10, we showed the number of TAD boundaries detected in chr17 and chr10 in the first column of the first and fourth rows. It is more clear that Hi-C results in fewer boundaries than C2c-predicted Micro-C. From the Venn figures in the first column, we can find that there are many more intersection boundaries between Micro-C and C2c-predicted Micro-C than between Micro-C and Hi-C.

**Figure 11:**
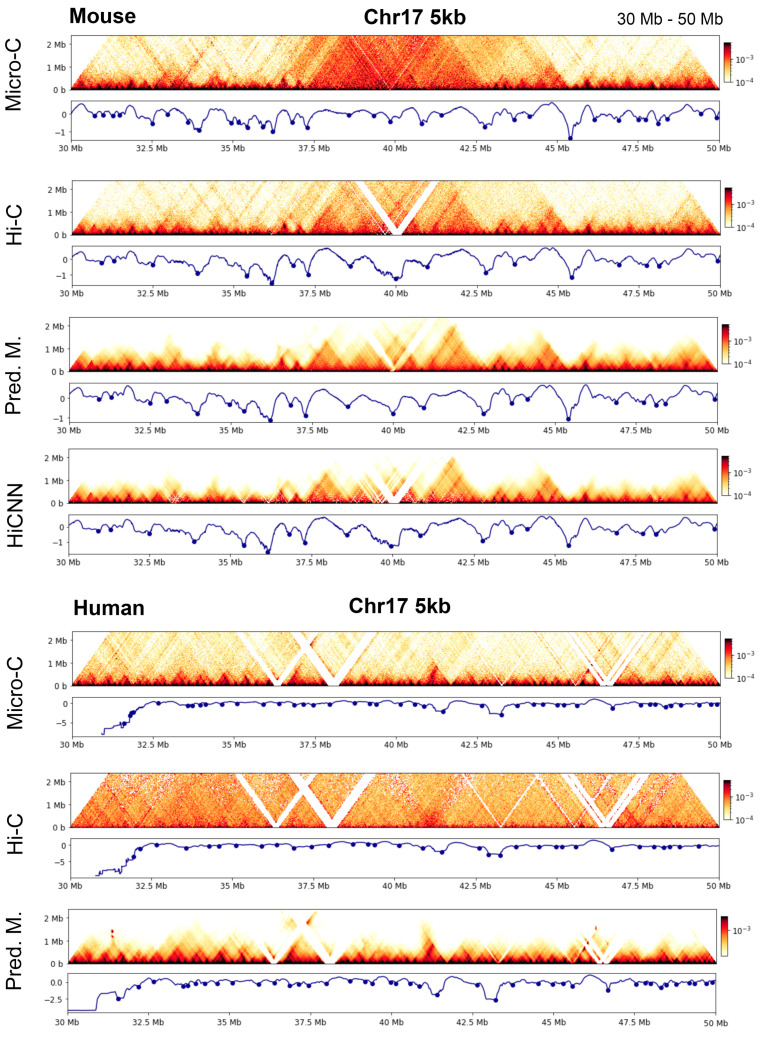
Heatmaps and the corresponding boundaries in the region (30 Mb–50 Mb) in 5 kb mouse and human data.

To illustrate these findings, in Figure 11, we present examples showing the TAD boundaries detected from a region in chromosome 17 of human and mouse genomes.

## 4. Discussion

In this study, we developed a new computational tool named C2c to predict high-resolution Micro-C contact matrices based on Hi-C. We designed a residual neural network to learn the mapping between Hi-C and real Micro-C contact matrices. We trained deep-learning models on mouse ES cells and then benchmarked these models on mouse and human ES cells.

From the C2c-predicted Micro-C contact matrices, the loop-calling tool can identify the loops that are ambiguous in Hi-C but are very evident in Micro-C. Our evaluation results indicate that C2c can significantly improve the ability to detect loops on contact maps. By analyzing the validity of the loops identified from the predicted Micro-C data, including comparing the number of consistent loops with those in real Micro-C loops, analyzing the statistical significance of the detected loops, identifying the quantity of enhancer–promoter matching loops, and cross-validation with ChIA-PET contact matrices, we proved that the loops identified from C2c-predicted Micro-C better match those from real Micro-C compared to Hi-C and can result in more loops that were not detected from real Micro-C.

Meanwhile, C2c-predicted Micro-C data result in more mutual TAD boundaries with real Micro-C compared to Hi-C. We also measured the insulation scores of the TAD boundaries and found that real Micro-C in general leads to shorter TADs compared to both Hi-C and C2c-predicted data.

From our evaluation results, we found that C2c can more significantly improve the loops than TAD boundaries, particularly for the Hi-C data, which have a high sequencing depth.

## Data Availability

C2c is freely available at http://dna.cs.miami.edu/C2c/ and https://github.com/zwang-bioinformatics/C2c/ (accessed on 21 April 2024).

## Supplementary Materials

The following supporting information can be downloaded at: https://www.mdpi.com/article/10.3390/genes15060673/s1. Figure S1: Heatmaps of the averaged contact maps of real Micro-C, Hi-C, and C2c predicted Micro-C that centered at the loops from real Micro-C data; Figure S2: Number of loops and enhancer-promoter matching loops that were identified from the real Micro-C, Hi-C, and C2c predicted Micro-C contact maps.
