# Supplementary material for: C2c: Predicting Micro-C from Hi-C
Source: Genes (Basel). 2024 May 23;15(6):673. doi: 10.3390/genes15060673 (PMC11203216; doi:10.3390/genes15060673)
Supplement: Supplementary file 1 [file genes-15-00673-s001.zip › genes-2999808-supplementary.pdf]

Supplementary file

C2c: Predicting Micro-C from Hi-C

Hao Zhu[1], Tong Liu[1] and Zheng Wang[1,*]

[1] Department of Computer Science, University of Miami, 330M Ungar Building, 1365 Memorial Drive, Coral Gables, FL, 33124-4245

*To whom correspondence should be addressed.

Contact: zheng.wang@miami.edu

Figures:

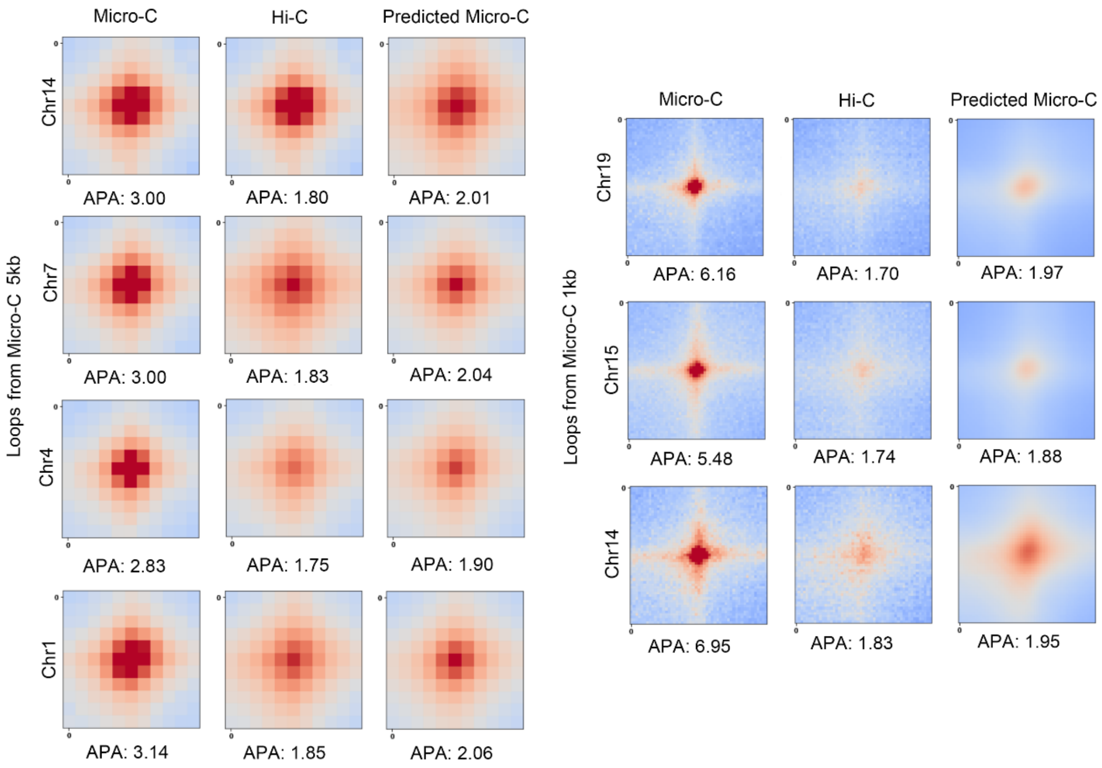

Figure S1. Heatmaps of the averaged contact maps of Micro-C, Hi-C, and C2c predicted Micro-C that centered at the loops from Micro-C data. APA score is shown at the bottom of each heatmap. The left four lines are from 5kb resolution, right three lines are from 1kb resolution.

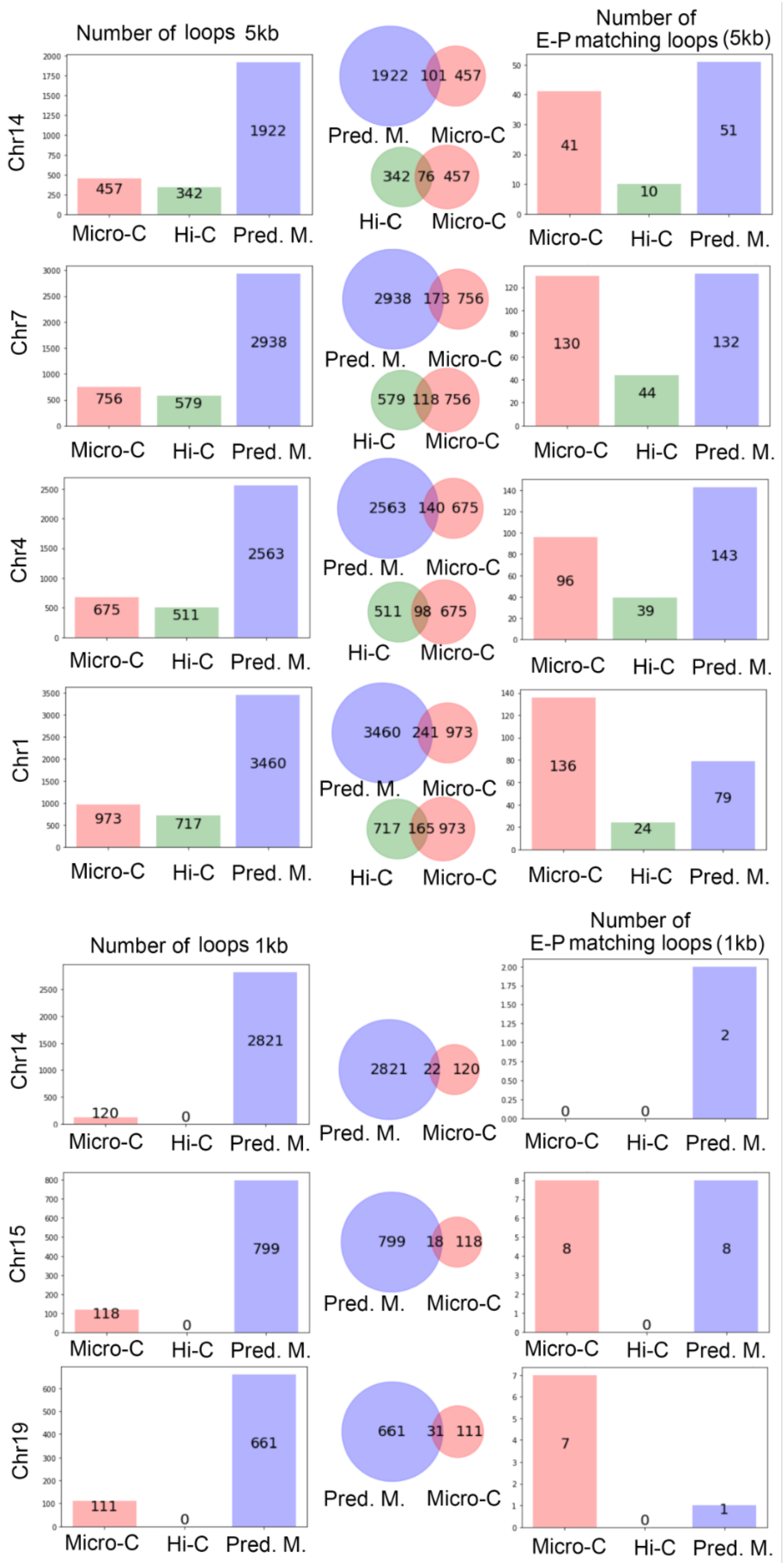
Number of loops 5kb
Number of E-P matching loops (5kb)
Chr14
457
342
1922
1922 101 457
Pred. M.
Micro-C
342 76 457
Hi-C
Micro-C
41
10
51
Chr7
756
579
2938
2938 173 756
579 118 756
130
44
132
Chr4
675
511
2563
2563 140 675
511 98 675
96
39
143
Chr1
973
717
3460
3460 241 973
717 165 973
136
24
79
Micro-C
Hi-C
Pred. M.
Number of loops 1kb
Number of E-P matching loops (1kb)
Chr14
120
0
2821
2821 22 120
0
0
2
Chr15
118
0
799
799 18 118
8
0
8
Chr19
111
0
661
661 31 111
7
0
1

Figure S2. Number of loops or enhancer-promoter matching loops that were identified from the real Micro-C, Hi-C, and C2c predicted Micro-C contact maps. The number of loops that both from Hi-C and Micro-C data, or from Micro-C and C2c predicted Micro-C data are shown as the intersection of two circles. The top four lines are from 5kb resolution, bottom three lines are from 1kb resolution.
